# Association of Ascending Aortic Aneurysm with NOX4 and miRNA 146a

**DOI:** 10.3390/genes17060709

**Published:** 2026-06-20

**Authors:** Recep Çalışkan, Osman Eren Karpuzoğlu, Fatma Hande Karpuzoğlu, Canan Küçükgergin, Kandemir Baş, Cevdet Uğur Koçoğulları

**Affiliations:** 1 Department of Cardiovascular Surgery, Edirne Sultan 1. Murat State Hospital, Edirne 22030, Turkey; 2 Department of Cardiovascular Surgery, Memorial Ataşehir Hospital, İstanbul 34750, Turkey; erenkarpuzoglu@yahoo.com; 3 Department of Medical Biochemistry, Istanbul Faculty of Medicine, Istanbul University, İstanbul 34093, Türkiye; handekarpuzoglu@yahoo.com (F.H.K.); ckucukgergin@yahoo.com (C.K.); 4 Department of Cardiovascular Surgery, Muğla Training and Research Hospital, Muğla 48000, Turkey; kandemirbas@yahoo.com; 5 Department of Cardiovascular Surgery, Dr. Siyami Ersek Chest, Heart and Vascular Surgery Training and Research Hospital, İstanbul 34668, Turkey; cevdetkocogullari@gmail.com

**Keywords:** ascending aortic aneurysm, NADPH oxidase 4, miR-146a

## Abstract

**Objective:** To evaluate the efficacy of NADPH oxidase 4 and miR-146a-5p in current treatment planning for ascending aortic aneurysms, independent of aortic diameter, and to develop protocols that will ensure the treatment of ascending aortic aneurysms, which pose a risk for aortic dissection, without complications. **Methods:** Patients who met the inclusion criteria and underwent surgery at Dr. Siyami Ersek Chest, Heart, and Vascular Surgery Training and Research Hospital for ascending aortic aneurysms and coronary artery disease between 2023 and 2024 were included in the study. This study was designed as a prospective study. Demographic, biochemical, radiological, and echocardiographic data were collected, and NOX4 mRNA and miR-146a-5p expressions were examined and compared in tissue samples. **Results:** The study was conducted on a total of 50 patients, with 25 patients in the aneurysm group and 25 patients in the control group. miR-146a-5p expression levels were found to be significantly decreased in the patient group compared to the control group (*p* = 0.001). When NOX4 mRNA expression levels were examined, no significant difference was found between the control and aneurysm groups. No correlation was found between NOX4 mRNA and miR-146a-5p levels (*p* = 0.764). When the relationship between ascending aorta diameter and both NOX4 mRNA and miR-146a-5p was examined, it was found that miR-146a-5p expression was negatively correlated with ascending aorta diameter (*p* = 0.036) and did not show a significant correlation with NOX4 mRNA levels (*p* = 0.318). A similar correlation was also found with ascending aorta length. The correlation of NOX4 mRNA and miR-146a-5p expression levels with age, gender, and ejection fraction was investigated separately. No significant correlation was found for all three variables. The optimum cut-off value to be used to separate the patient group from the control group using miR-146a-5p expression levels, as well as the sensitivity and specificity of miR-146a-5p expression levels when this cut-off value was used, was calculated using an ROC curve. Specificity for miR-146a-5p expression was found to be 88%, and sensitivity was found to be 66%. **Conclusions:** The study found promising results indicating that NOX4, shown to be a determinant of vascular oxidative stress, is not involved in the development of ascending aortic aneurysms; however, miR-146a-5p, which functions in the regulation of many inflammatory responses, including the regulation of NOX4 expression, may help prevent the development of ascending aortic aneurysms. Further studies aimed at elucidating the genetic and biochemical processes involved in aneurysm development suggest that miR-146a-5p could be a therapeutic target for preventing aneurysms.

## 1. Introduction

The term aneurysm is defined as a segmental dilatation of a vessel with at least a 50% increase in the expected normal diameter. Thoracic aortic aneurysms, including ascending aortic aneurysms, are associated with aging and with genetic and biochemical changes in the aortic wall protein components that alter the response of the aorta to biomechanical stimuli [1]. Recently, epigenomic, transcriptomic, proteomic, and metabolomic techniques have been used to determine the biological basis and etiological mechanisms of thoracic aortic aneurysms [2].

Reactive oxygen species (ROS) serve as vital mediators in the pathogenesis of various cardiovascular diseases, notably hypertension, atherosclerosis, diabetes, and heart failure. The generation of ROS within the vascular bed is attributed to several enzymatic systems with distinct localization patterns, including xanthine oxidase, cytochrome P450 monooxygenase, endothelial nitric oxide synthase, and nicotinamide adenine dinucleotide phosphate (NADPH) oxidase [3]. Of these, NADPH oxidases have been identified as the predominant source of ROS in the vessel wall, primarily due to their regulatory influence over the activation and impairment of other enzymes [4]. Various isoforms, specifically NOX1, NOX2, NOX4, and NOX5, are distributed across diverse vascular components, ranging from endothelial and smooth muscle cells to fibroblasts and perivascular adipocytes [5]. NOX4 functions as a multifaceted enzyme that modulates angiogenesis, cellular defense mechanisms, and antioxidant pathways, as well as inflammatory responses and matrix remodeling. Extensive research has explored the interplay between these specific enzymatic systems and the development of hypertension, atherosclerosis, and altered vascular performance [6,7,8].

It has been established that microRNAs (miRs) are essential regulators of oxidative stress pathways in various vascular pathologies [9]. A diverse array of miRNAs intermediate- and long-term patient outcomes [10]. Existing literature indicates a distinct expression profile in thoracic aortic dissection, characterized by the upregulation of miR-491-3p, miR-338-5p, miR-433, miR-183, miR-553, and miR-30c, contrasted by the suppression of miR-24, miR-143, miR-22, miR-93, and miR-145 [11]. Regarding the miR-146 family, Taganov et al. [12] first characterized its genomic organization and regulatory mechanisms in 2006 and simultaneously confirmed the presence of its human homolog.

Oxidative stress is a key driver of vascular remodeling and degeneration, contributing to inflammation, extracellular matrix degradation, and vascular smooth muscle cell dysfunction. Among the major enzymatic sources of reactive oxygen species (ROS), NADPH oxidase 4 (NOX4) plays a central role in vascular redox homeostasis and has been implicated in the pathogenesis of several cardiovascular diseases, including ascending aortic aneurysm (AsAA). Increased NOX4 activity promotes ROS generation, leading to the activation of pro-inflammatory signaling pathways and the progressive structural deterioration of the aortic wall. MicroRNAs have emerged as important post-transcriptional regulators of genes involved in oxidative stress and vascular homeostasis. In particular, miR-146a-5p is a well-characterized anti-inflammatory microRNA that suppresses NF-κB signaling by inhibiting IRAK1 and TRAF6. Recent in silico analyses and experimental studies suggest that miR-146a-5p may also regulate NOX4 expression, indicating a potential role in modulating oxidative stress-mediated vascular injury. Based on these findings, we hypothesized that the downregulation of miR-146a-5p may result in the derepression of NOX4, leading to enhanced oxidative stress and activation of inflammatory and matrix-degrading pathways and ultimately contributing to AsAA development. Therefore, we investigated the expression profiles of miR-146a-5p and NOX4 in aneurysmal and non-aneurysmal ascending aortic tissues and evaluated their potential regulatory relationship.

There is no study in the literature investigating the relationship between miR-146a-5p and ascending aortic aneurysm. Research on the discovery of new miRNAs and protein targets involved in the protection against or modulation of oxidative stress is essential for developing miRNA-based treatment strategies.

It has been observed that more than 60% of patients presenting with acute type A aortic dissection had an aortic size below the surgical treatment size of 55 mm for an aortic aneurysm [13]. Even if dissected aortas do not reach the limit for elective intervention, dissections occur on the basis of an aneurysm; therefore, an aneurysm is a significant indicator for the development of dissection [14]. In addition to the surgical treatment of aneurysms, there is a need to elucidate the factors that cause aneurysms and to prevent their development by addressing these causes. Despite growing evidence of microRNA (miRNA) regulation in vascular disease, the potential post-transcriptional interaction between NADPH oxidase 4 (NOX4) and microRNA-146a has not been well studied in the context of ascending aortic aneurysms. In this study, NOX4, a primary source of vascular reactive oxygen species (ROS), and miR-146a-5p, an essential regulator of inflammatory signaling, were selected to investigate a potential regulatory relationship in this disease. In silico miRNA–mRNA interaction analysis was conducted using the DIANA-microT 2023 algorithm, which predicted a possible binding site for miR-146a-5p within the 3′ untranslated region of NOX4. Based on this bioinformatic evidence, the present study investigated the relationship between NOX4 expression and miR-146a-5p expression levels in patients with ascending aortic aneurysms.

## 2. Materials and Methods

A priori sample size calculation was performed using G*Power (version 3.1.9.4). The analysis was based on an independent two-group comparison, assuming a moderate effect size (Cohen’s d = 0.6), a significance level (α) of 0.05, and a statistical power of 80%. Based on these assumptions, the minimum required sample size was estimated at 24 participants per group. The expected effect size was determined based on previously published gene expression studies in vascular tissues reporting moderate differences between aneurysmal and non-aneurysmal aortic samples.

A total of 50 patients who underwent surgery in our hospital between 2023 and 2024 were included in the study. The sample consisted of 25 patients who underwent surgery for an ascending aortic aneurysm and 25 patients who underwent coronary artery bypass surgery to serve as the control group. Patients who underwent ascending aorta replacement in addition to aortic insufficiency or coronary artery bypass graft surgery were also included in the aneurysm group. The study protocol was approved by the Istanbul Haydarpaşa Numune Training and Research Hospital Clinical Research Ethics Committee (HNEAH-KAEK; Decision Date: 17 April 2023 and Decision No: 2023/64) and conducted in accordance with the Declaration of Helsinki. Prior to the procedure, patients were informed about the study, and informed consent was obtained from all patients through completed consent forms.

**Obtaining, Transferring and Storing Tissues**

After anesthesia induction and before the surgical intervention began, 20 cc of venous blood was collected from patients with aortic aneurysms and multivessel coronary bypass surgery into biochemistry tubes via a central venous catheter. The samples were centrifuged at 4000 rpm for 10 min. Serum samples were then separated from the tubes and stored at −80 degrees until biochemical studies were performed. Aortic tissues were removed perioperatively from patients with an aortic aneurysm and multivessel coronary bypass surgery. Ascending aortic tissues from coronary bypass surgery patients were obtained from the punch-hole tissues for proximal anastomosis. The removed aortic tissues were kept in RNAlater solution at +4 degrees for 24 h. Then, the tissues were transferred to a −80° deep freezer on dry ice.

Total RNA was extracted from tissue using the miRNeasy Tissue/Cells Advanced kit (Qiagen, Germantown, MD, USA; Cat. No. 217684) according to the manufacturer’s instructions. Complementary DNA (cDNA) synthesis was performed using the miRCURY LNA^TM^ RT Kit (Qiagen, USA; Cat. no. 339346) and the RT^2^ First Strand Kit (Qiagen, Catalog no: 330404).

Expression levels of miR-146a-5p and mRNA NOX4 were quantified using a Rotor-Gene Q real-time PCR system (Qiagen, USA). Primers were purchased from Qiagen Technologies: miRCURY LNA^TM^ miRNA PCR Assay U6 (Cat. no. YP02119464), miRCURY LNA^TM^ miRNA PCR Assay hsa-miR-146a-5p (Cat. no. YP000204688), RT^2^ mRNA qPCR Assay (NOX4) (Cat. no. PPH06078A-200), and RT^2^ lncRNA qPCR Assay (GAPDH) (Cat. no. 330001PPH00150F). For endogenous controls, GAPDH was used as the reference for NOX4, which is thought to be expressed equally across all tissues, and U6 was used as the reference for the miRNA. Using the obtained CT values, the ΔΔCT ((ΔΔCT = (CT value of endogenous reference ncRNA of aneurysmal tissue)—(CT value of target ncRNA of normal tissue—CT value of endogenous reference ncRNA of normal tissue)) value of the samples was calculated. The “fold change”, which is the change in expression level in aneurysmal and normal tissue, was calculated with the 2^−ΔΔCT^ formula.

**In silico miRNA–mRNA Interaction Analysis**

In silico prediction of miRNA–mRNA interactions was performed using the DIANA-microT 2023 algorithm ([15] Spyros Tastsoglou, Athanasios Alexiou, Dimitra Karagkouni, Giorgos Skoufos, Elissavet Zacharopoulou, and Artemis G. Hatzigeorgiou; DIANA-microT 2023: including predicted targets of virally encoded miRNAs, *Nucleic Acids Research*, DOI:10.1093/nar/gkad283). The analysis identified potential miR-146a-5p binding sites within the NOX4 transcript. DIANA-microT evaluates candidate interactions based on sequence complementarity, binding type, and evolutionary conservation. A high-confidence 9-mer binding site for miR-146a-5p was identified within the 3′-untranslated region of the NOX4 transcript (positions 2165–2184), with a microT score of 0.92 (scale 0–1, where higher scores indicate stronger predicted interactions).

**Statistics**

SPSS version 26.0 (SPSS, Chicago, IL, USA) and GraphPad Prism version 9.5.1 were used for statistical analyses and graphical presentations. The conformity of the data to normal distribution was assessed using the Kolmogorov–Smirnov test, supported by visual inspection of histograms and Q–Q plots. Comparisons of ncRNA expression levels between aneurysmal and non-aneurysmal tissues were performed using the Mann–Whitney U test. Differences in NOX4 and miR-146a-5p expression levels according to histopathological and clinical features that did not show normal distribution were also assessed using the Mann–Whitney U test. Categorical variables were compared using Fisher’s exact test. Given the significant differences in the prevalence of diabetes mellitus (DM) and chronic obstructive pulmonary disease (COPD) between the control and ascending aortic aneurysm (AsAA) groups, these variables were treated as potential confounders. Therefore, analysis of covariance (ANCOVA) was performed to compare NOX4 and miR-146a-5p expression levels between groups, with DM and COPD included as covariates. Correlations between variables were evaluated using Spearman’s rank correlation analysis. Receiver operating characteristic (ROC) curve analysis was used to determine the cut-off values and the sensitivity and specificity of ncRNA expression levels in aneurysmal tissue. Statistical significance was assessed at the 95% confidence level (*p* < 0.05).

## 3. Results

Preoperative contrast-enhanced CT angiographic images of the patients in the aneurysm group were examined. The diameters of the aortic annulus, sinus of Valsalva, and ascending aorta at the pulmonary bifurcation were measured. The lengths of the ascending aorta from the aortic annulus to the innominate artery were measured. The mean aortic annulus diameter in the aneurysm group was found to be 24.5 ± 2.2 mm, the sinus of Valsalva diameter was 45.2 ± 7.4 mm, the ascending aorta diameter was 51.0 ± 6.0 mm, and the ascending aorta length was 115.1 ± 10 mm. The demographic and clinical characteristics of the patients included in the study are shown in Table 1.

**Figure 1:**
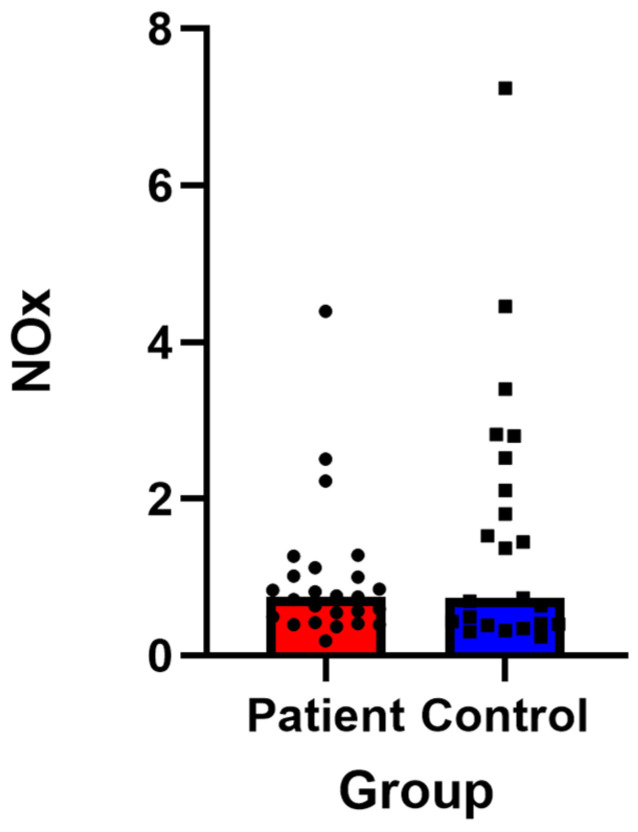
NOX4 mRNA expression in the control and aneurysm groups.

**Figure 2:**
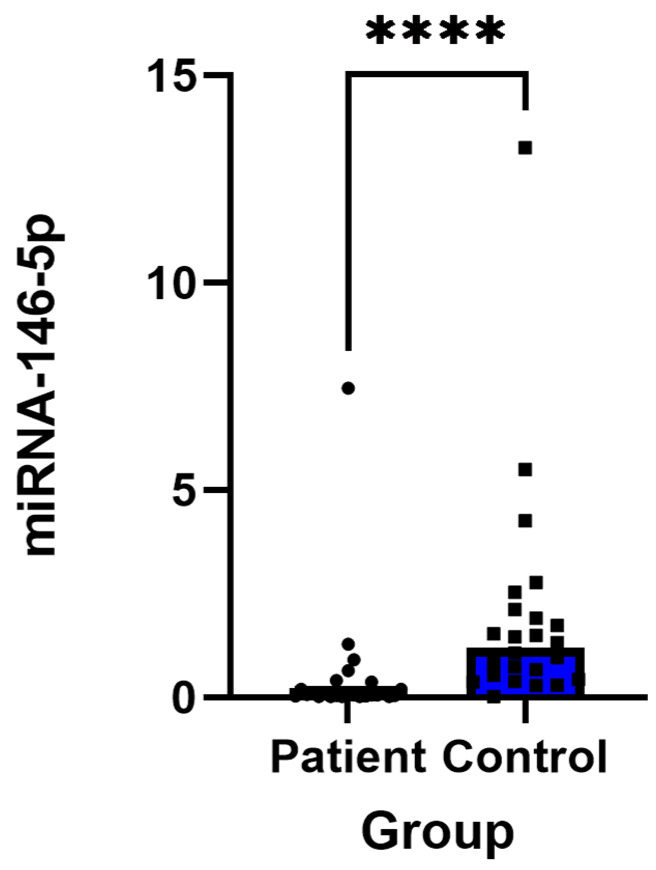
miR-146a expression in the control and aneurysm groups.

miR-146a-5p expression levels in the aneurysm group were significantly lower than those in the control group (*p* = 0.001). When NOX4 mRNA expression levels were examined, no significant difference was observed between the control and aneurysm groups (*p* = 0.549) (Table 2, Figure 1 and Figure 2). miR-146a-5p downregulation remained statistically significant after adjustment for DM and COPD using ANCOVA (adjusted *p* = 0.013).

The correlation between NOX4 mRNA and miR-146a-5p expression levels in the aneurysm and control groups was assessed using the Spearman correlation test. The analysis revealed no significant correlation between NOX4 mRNA and miR-146a-5p levels (**Spearman’s** *ρ* = **0.043**, *p* = 0.764; 95% CI: −0.254 to 0.336). When exploring the relationship between ascending aortic dimensions and gene expressions, a significant negative correlation was identified between miR-146a-5p expression and ascending aortic diameter (*ρ* **= −0.312**, *p* = 0.036; 95% CI: −0.537 to −0.022). In contrast, no correlation was detected between ascending aortic diameter and NOX4 mRNA levels (*ρ* = **−0.147**, *p* = 0.318; 95% CI: −0.414 to 0.143). Additionally, a significant negative correlation was observed between miR-146a-5p expression and ascending aorta length (*ρ* = **−0.306**, *p* = 0.029; 95% CI: −0.546 to −0.034). Conversely, no significant correlation was found between ascending aorta length and NOX4 mRNA levels (*ρ* = **−0.228**, *p* = 0.120; 95% CI: −0.481 to 0.060).

Patients were grouped into those with and without aortic valve insufficiency. The relationship between NOX4 mRNA and miR-146a-5p expressions and aortic valve insufficiency was analyzed. miR-146a-5p expression was significantly lower in the patient group with aortic valve insufficiency (*p* = 0.019), whereas NOX4 mRNA expression showed no statistically significant difference (*p* = 0.601).

A Spearman correlation test was performed for the correlation between age, gender, EF value, and NOX4 mRNA and miR-146a-5p expression levels in the aneurysm and control groups. In the analysis, no significant correlations were found between age and NOX4 mRNA (ρ = −0.016, *p* = 0.915; 95% CI: −0.306 to 0.278) or miR-146a-5p (ρ = 0.071, *p* = 0.625; 95% CI: −0.214 to 0.345). Similarly, gender showed no correlation with NOX4 mRNA (ρ = −0.061, *p* = 0.681; 95% CI: −0.347 to 0.235) or miR-146a-5p levels (ρ = −0.043, *p* = 0.769; 95% CI: −0.328 to 0.249). Finally, no significant correlation was observed between ejection fraction (EF) and NOX4 mRNA (ρ = −0.155, *p* = 0.167; 95% CI: −0.467 to 0.095) or miR-146a-5p levels (ρ = −0.264, *p* = 0.068; 95% CI: −0.513 to 0.029).

**Figure 3:**
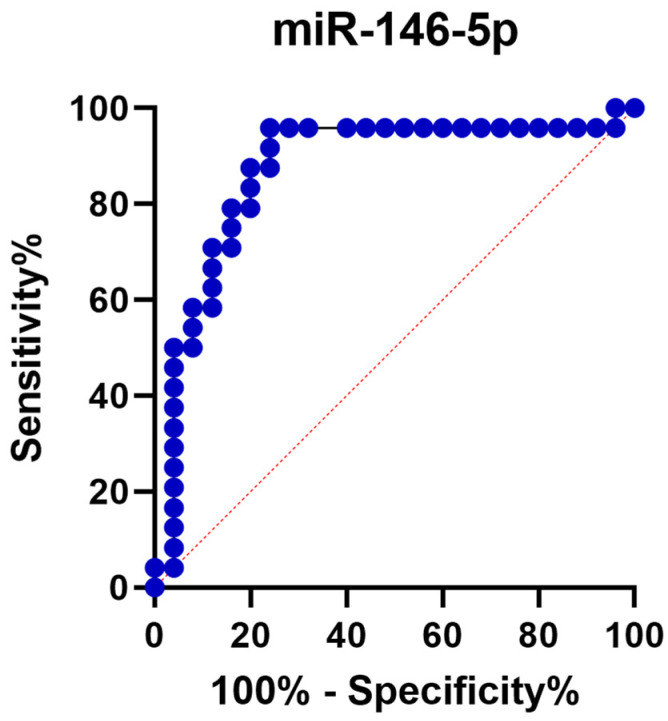
miR-146a expression ROC (receiver operating characteristic) curve. ***AUC:* [0.870]** (95% CI: **[0.75–0.98]**; *p* < 0.001).

The receiver operating characteristic (ROC) curve analysis was performed to evaluate the diagnostic performance of miR-146a-5p in distinguishing aneurysm patients from controls. The area under the curve (AUC) was found to be 0.870 (95% CI: 0.75–0.98, *p* < 0.001). The optimal cut-off value of miR-146a-5p expression was determined as 0.70 using the Youden index. At this threshold, the sensitivity was 66%, and the specificity was 88% (Figure 3).

## 4. Discussion

miRNAs have been reported to play an essential role in regulating oxidative stress responses in vascular diseases [9]. Studies show that miRNAs can be used for early diagnosis of aortic aneurysms, for medium- and long-term prognosis, and for identifying therapeutic targets [10,11,16]. Venkatesh et al. [17] investigated the activities of miRNAs in aortic aneurysms. The study showed that miR-221, especially in thoracic aortic aneurysms, and miR-146a-5p in abdominal aortic aneurysms, increased. It was emphasized that the elevation of miR146a in abdominal aneurysms may be associated with increased inflammatory activity. In the same study, miR146a levels did not differ significantly between the thoracic aortic aneurysm group and the control group. The lack of a significant difference may be due to the control group being selected from patients with heart failure who underwent transplantation surgery and to increased inflammatory changes in heart failure, which may have eliminated the difference by increasing miR146a levels. Mingfang Liao et al. [11] found that miR-146a-5p was further decreased in dissection patients than in aneurysm patients in their study based on a microRNA profile comparison between thoracic aortic dissection and normal thoracic aortas. In the study by HL Zhang et al. [18] on miR-146a-5p expression in patients with intracranial aneurysms, higher miR-146a-5p expression was observed in the aneurysm group. It was also shown that miR-146a-5p level was an independent risk factor, and high levels were associated with shorter survival. These findings suggest that miR-146a-5p regulation is disease-specific and influenced by both aneurysm location and the underlying inflammatory state.

In our study, miR-146a-5p levels were significantly lower in the aneurysm group than in the control group (*p* < 0.001). DM was more prevalent in the control group, while COPD was more frequent in the aneurysm group. Both conditions are associated with chronic inflammation, oxidative stress, and altered miRNA profiles. However, miR-146a-5p downregulation remained significant after adjustment for DM and COPD (adjusted *p* = 0.013), indicating an aneurysm-specific regulatory mechanism.

Our research yielded data suggesting that miR-146a-5p may have a protective effect against the development of ascending aortic aneurysms. We believe this is achieved through its suppressive effect on pro-inflammatory processes. Correlations with ascending aortic diameter, length, and aortic valve insufficiency were also examined, revealing that miR-146a-5p was downregulated. This result provides valuable data for elucidating the biochemical processes underlying the development of ascending aortic aneurysms. It suggests that a new horizon may be opened in the diagnosis, monitoring, and treatment of this disease, which is currently treated solely by changes in aortic diameter. We determined the optimal threshold for differentiating the patient group from the control group based on miR-146a-5p expression levels. Using this value, we found that the specificity for miR-146a-5p expression was 88%, and the sensitivity was 66%. In this context, although multiple independent parameters and inflammatory processes affect the expression of miR-146a-5p, our study demonstrated its effectiveness in the development of ascending aortic aneurysms, providing important data for a better understanding of the biochemical processes underlying sporadic ascending aortic aneurysms. There are no human studies in the literature demonstrating the effectiveness of NOX4/miR-146a-5p in sporadic ascending aortic aneurysms. Our study is therefore the first of its kind.

It has been previously shown that in states of increased pro-atherosclerotic risk, such as hypercholesterolemia, NOX4 may protect against endothelial dysfunction and atherosclerosis [19]. Schröder et al. [8] showed that endogenous NOX4 is a constitutive endothelial H_2_O_2_ generator and a protective NADPH oxidase that positively affects vascular function. However, another study showed that NOX4 has a detrimental effect on the cardiovascular system in various animal models [6]. Previous studies have concluded that increased NOX4 presence indicates increased oxidative stress [3,19,20,21]. It has been observed that NOX4 is involved in the development of certain cardiovascular diseases. The main ones are: atherosclerosis, hypertension, primary pulmonary hypertension, fibrosis, stroke, and heart failure [7]. There are a few studies, especially in cell culture and animal models, showing that NOX4 is effective in aneurysm development [22,23,24]. To our knowledge, this is the first human study to evaluate the role of NOX4 in the pathogenesis of sporadic ascending aortic aneurysm. Our findings indicate that NOX4 is not independently associated with the development of sporadic ascending aortic aneurysm in humans.

Our study found no significant difference in NOX4 mRNA expression between the aneurysm and control groups. Correlation with aortic diameter, length, and aortic insufficiency was also not significant. Therefore, while our study demonstrates that NOX4 is not associated with aneurysm development in the vascular bed, we consider the measurement of only mRNA expression and the lack of protein quantification (e.g., Western blot or ELISA), along with post-transcriptional regulation, to be a limitation of our study.

Although in silico analysis predicted a binding site for miR-146a-5p in the NOX4 3’-UTR, we observed no significant correlation between their expression levels (*p* = 0.764). This discrepancy may be explained by several factors: (1) miRNAs often regulate protein translation without affecting mRNA levels, whereas we measured only mRNA expression; (2) computational predictions indicate potential interactions but do not guarantee functional regulation in vivo; and (3) the complex inflammatory and oxidative stress networks in aneurysm pathogenesis likely involve multiple redundant regulatory mechanisms that may mask individual miRNA–target correlations. Protein-level analyses and functional validation are needed to clarify this potential regulatory relationship between miR-146a-5p and NOX4 in ascending aortic aneurysm.

Our study has several limitations that should be acknowledged:

**Sample Size and Subgroup Analysis:** The relatively small sample size (n = 50) warrants validation in larger multicenter cohorts. Furthermore, the power of our subgroup analyses—such as those involving patients with aortic valve insufficiency—was limited by the lack of specific patient counts (n) for each category, potentially restrict the generalizability of these findings.

**mRNA vs. Protein Quantification:** A primary limitation is that we measured only mRNA expression levels for NOX4. Given that microRNAs, including miR-146a-5p, can inhibit protein translation without necessarily inducing mRNA degradation, measuring transcript levels alone—without protein-level validation via Western blot, ELISA, or immunohistochemistry—prevents definitive conclusions regarding the actual enzymatic activity of the NOX4 pathway.

**Diagnostic Performance and Clinical Translation:** While miR-146a-5p demonstrated high specificity (88%), its moderate sensitivity of 66% implies a notable rate of false-negative results. Consequently, miR-146a-5p should be viewed as a potential component of a broader diagnostic panel rather than a standalone clinical biomarker, and our enthusiasm for its immediate clinical translation is tempered by this finding. Furthermore, while the present study utilized surgically obtained tissue samples, future research focusing on the expression of miR-146a-5p in circulating plasma or serum is warranted. Investigating these non-invasive sources could provide a more clinically applicable and feasible approach for the routine screening and longitudinal monitoring of patients at risk for ascending aortic aneurysms.

**Confounding Factors:** Significant differences in the prevalence of diabetes mellitus (DM) and chronic obstructive pulmonary disease (COPD) were observed between the groups. Although we utilized ANCOVA to adjust for these variables, the lack of further sensitivity analyses—such as propensity score matching or stratified analysis—means that the potential influence of these chronic conditions on miRNA profiles cannot be entirely excluded. In addition, the use of patients undergoing coronary artery bypass grafting as the control group may have introduced bias due to underlying atherosclerotic disease and systemic inflammatory activation, which could potentially affect oxidative stress-related pathways and miRNA expression profiles.

**Functional Validation:** Although in silico analysis predicted a strong binding site for miR-146a-5p in the NOX4 3’-UTR (microT score: 0.92), we did not observe a significant correlation between their expression levels. The lack of experimental validation, such as luciferase reporter assays or AGO2-RIP, remains a limitation in confirming this predicted interaction.

**Study Scope:** Finally, the inclusion of patients with acute aortic dissection in future research would provide more comprehensive insights into the progression from aneurysm to dissection.

## 5. Conclusions

In our study, NOX4, an essential source of oxidative stress in the vascular bed, did not significantly affect the development of ascending aortic aneurysms. No significant correlation was found between miR-146a-5p and NOX4 in aneurysm development. In ascending aortic aneurysms, miR-146a-5p was downregulated, suggesting that miR-146a-5p may have a preventive effect on the development of ascending aortic aneurysms. The cut-off value for miR-146a-5p was determined. miR-146a-5p offers new insights into the biochemical processes involved in the development of sporadic ascending aortic aneurysms, opening new horizons for improving current follow-up and treatment strategies for the disease.

## Figures and Tables

**Table 1:** Demographic and clinical characteristics of the study population.

		Control Group (N:25)	Aneurysm Group (N:25)	*p* Value
Age (mean ± mean deviation)		62.7 ± 9.9	58.2 ± 10.2	0.177
Gender (%)	Female	7 (%28)	7 (%28)	
	Male	18 (%72)	18 (%72)	1.000
Hypertension (%)		25 (%100)	23 (%92)	0.149
Hyperlipidemia (%)		10 (%40)	10 (%40)	1.000
Diabetes Mellitus (%)		15 (%60)	6 (%24)	0.010
COPD (%)		0 (%0)	5 (%20)	0.018
Chronic Kidney Disease (%)		6 (%24)	3 (%12)	0.269
Atrial Fibrillation		0 (%0)	2 (%8)	0.149
Body Mass Index (kg/m^2^) (**mean ± SD**)		28.6 ± 4.6	28.6 ± 4.0	0.992
**Body Surface Area (m^2^)** (**mean ± SD**)		1.88 ± 0.17	1.95 ± 0.20	0.240

**Table 2:** NOX4 mRNA and miR-146a expression levels in the control and aneurysm groups.

	Control Group	Aneurysm Group	*p* Value
**NOX4 mRNA** Median (Min–Max)	0.73 (0.24–7.24)	0.75 (0.19–4.40)	0.549
**miR-146a** Median (Min–Max)	1.19 (0.01–13.26)	0.05 (0.01–7.46)	<0.001

## Data Availability

The raw data supporting the conclusions of this article will be made available by the authors on request.

## Abbreviations

ROS	Reactive oxygen species
NADPH	Nicotinamide adenine dinucleotide phosphate
NOX	NADPH oxidase
RNA	Ribonucleic acid
miRNA	microRNA
miR	microRNA
